# Interdisciplinary neurovascular networks in Germany: update 2025

**DOI:** 10.1186/s42466-025-00418-8

**Published:** 2025-08-22

**Authors:** Tobias A. Wagner-Altendorf, Konrad Sieb, Ansgar Berlis, Arnd Dörfler, Dorothee Mielke, Christoph Groden, Erdem Güresir, Gerhard F. Hamann, Olav Jansen, Jürgen Meixensberger, Oliver Müller, Darius G. Nabavi, Tobias Neumann-Haefelin, Martin Ossenbrink, Jan Regelsberger, Georg Royl, Hartmut Vatter, Werner Weber, Nils Werring, Jens Minnerup, Joachim Röther

**Affiliations:** 1 Department of Neurology, University Hospital Schleswig-Holstein, Ratzeburger Allee 160, 23538 Lübeck, Germany; 2 LGA InterCert GmbH, TÜV Rheinland Group, Nuremberg, Germany; 3 Department of Diagnostic and Interventional Neuroradiology, University Hospital Augsburg, Augsburg, Germany; 4 Department of Neuroradiology, Friedrich‑Alexander University of Erlangen‑Nuremberg, University Hospital Erlangen, Erlangen, Germany; 5 Department of Neurosurgery, University Hospital Augsburg, Augsburg, Germany; 6 Department of Neuroradiology, University Medical Center Mannheim, Medical Faculty Mannheim, University of Heidelberg, Mannheim, Germany; 7 Department of Neurosurgery, University Hospital Leipzig, Leipzig, Germany; 8 Department of Neurosurgery, University Hospital Bonn, Bonn, Germany; 9 Department of Neuroradiology, University Hospital Schleswig-Holstein, Kiel, Germany; 10 Department of Neurosurgery, Dortmund Hospital, Dortmund, Germany; 11 Department of Neurology and Neurological Rehabilitation, Günzburg Hospital, Günzburg, Germany; 12 Department of Neurosurgery, Diako Krankenhaus Flensburg, Flensburg, Germany; 13 Department of Neurology, Fulda Hospital, Fulda, Germany; 14 Department of Diagnostic and Interventional Radiology, Neuroradiology and Nuclear Medicine, Knappschaft Kliniken University Hospital, Bochum, Germany; 15 Department of Neurology, Vivantes Klinikum Neukölln, Berlin, Germany; 16 Department of Neurology, Asklepios Hospital Altona, Hamburg, Germany

**Keywords:** Neurovascular networks, Stroke care, Thrombectomy, Thrombolysis, Carotid endarterectomy, Carotid stenting, Intracranial aneurysm

## Abstract

**Background:**

Neurovascular networks (NVNs) in Germany are supra-regional care structures for patients with neurovascular diseases. Each NVN consists of a tertiary care center serving as the coordinating center—in some cases, two or three coordinating centers—and at least three partner hospitals. Since 2018, 19 neurovascular networks (NVNs) have been audited and certified. NVNs play a crucial role in stroke care in Germany, as first described and quantified in 2020.

**Methods:**

The present article provides an update on interdisciplinary NVNs in Germany and outlines recent developments in neurovascular patient care. Audit reports from 19 NVNs, certified between 2021 and 2024, were analyzed, and compared to previously reported data from 2017 to 2019. Additionally, structural and quality-related parameters for coordinating centers and partner hospitals were compared.

**Results:**

The number of NVNs increased from 15 to 19, with approximately 120,000 from an estimated 262,000 neurovascular patients in Germany now treated annually in certified NVN hospitals. In particular, annual thrombectomy rates at coordinating centers have increased over-proportionally (> 4400, as compared to previously < 2500), and surgical treatments for intracerebral hemorrhages have also increased. Process times—door-to-needle and door-to-groin times—remained stable or exhibited slight increases. Substantial variability was observed among NVN partner hospitals regarding procedural volumes.

**Conclusions:**

The treatment of patients with neurovascular diseases in Germany has expanded considerably within certified NVN hospitals in recent years. The NVNs ensure comprehensive, high-quality stroke care nationwide.

## Introduction

**Fig. 1:**
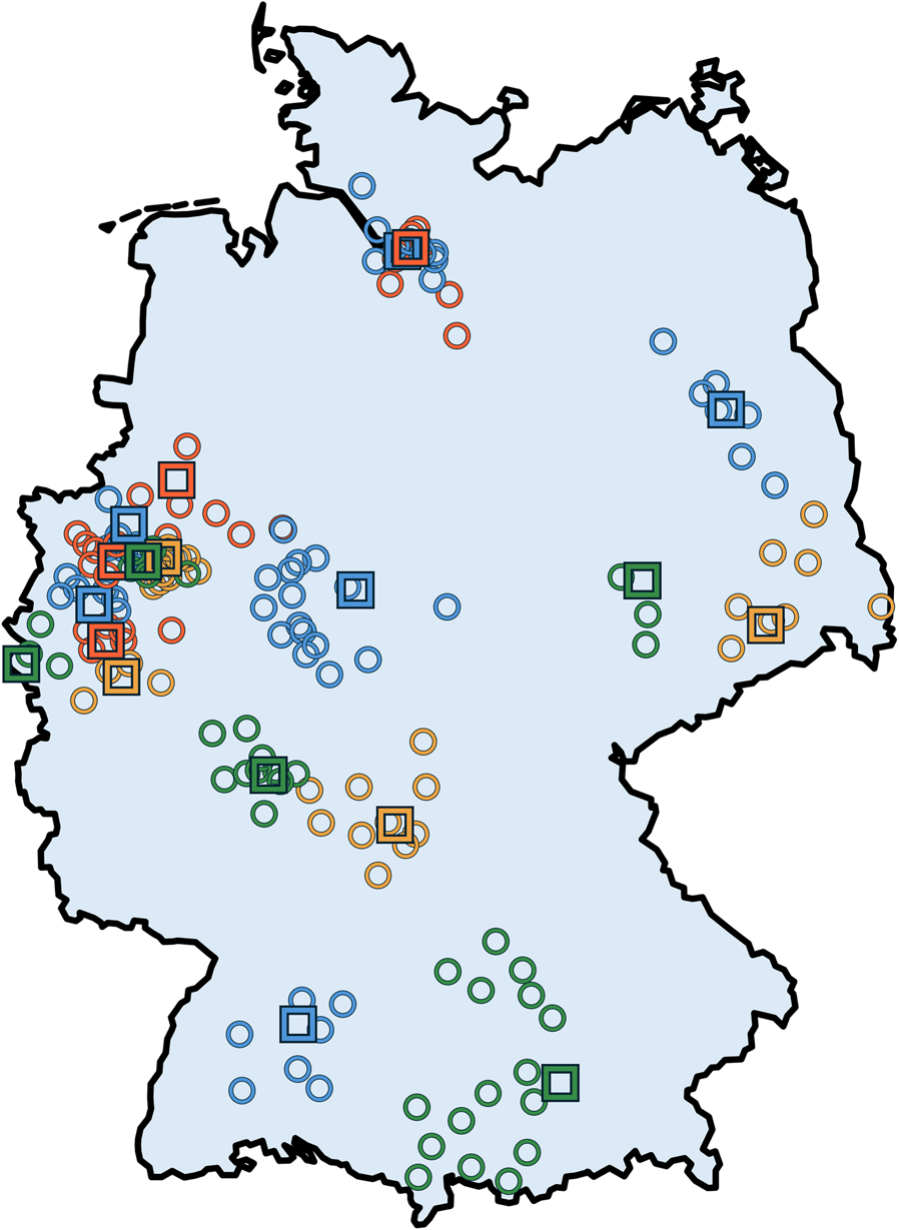
Map of Germany, showing the distribution of 19 certified neurovascular networks (NVNs). Coordinating centers are marked by squares, partner hospitals are shown as circles; color-coded by NVN affiliation

Neurovascular networks (NVNs) comprise at least one tertiary care center as coordinating center and additional partner hospitals. NVNs aim to improve the interdisciplinary, high-quality treatment for patients with neurovascular diseases, including rare entities, through specialized infrastructures [4, 12]. Following a pilot phase from 2012 to 2017, 19 NVNs have been audited and certified in Germany since 2018 by the national societies of neurology, neurosurgery, and neuroradiology. Requirements for coordinating centers include treating more than 1,000 stroke patients annually, maintaining a stroke unit with at least 12 beds, and operating independent departments of neurology, neurosurgery, neuroradiology, cardiology, and vascular surgery (see current certification guidelines at https://www.dsg-info.de/stroke-units-neurovaskulaere-netzwerke/zertifizierungsantraege-kriterien/ for a full list of requirements). Figure 1 illustrates the distribution of NVNs in Germany, highlighting coordinating centers and partner hospitals.

The formation of NVNs was substantially driven by major advancements in vascular neurology over recent decades, and a need for multidisciplinary cooperation due to several new medical, interventional, and surgical treatment options, including their potential combinations. Consecutively, a three-level care structure was implemented: regional stroke unit, supra-regional stroke unit, and NVN, with regional stroke units equating to European Stroke Organisation (ESO) stroke units, and supra-regional stroke units equating to ESO stroke centres ([8, 14], see current ESO certification guidelines at https://eso-certification.org/application/eso-certification-application-forms/). These structures ensure optimal treatment for patients with ischemic stroke, subarachnoid hemorrhage, intracerebral hemorrhage, vascular malformation, and extracranial and intracranial artery stenosis. The widespread adoption of mechanical thrombectomy in 2015, in particular, necessitated structured protocols and standardized referral routes to ensure timely access for all eligible patients [3, 7, 11]. Similarly, standardized protocols for the treatment of intracerebral and subarachnoid hemorrhages, as well as for the elective management of various arteriovenous malformations (such as aneurysms, fistulas, angiomas, and cavernomas), have become increasingly necessary. Given the rarity and complexity of these conditions, centralizing their treatment in specialized centers seems reasonable. Röther et al. [12] provided an assessment and quantification of the medical services, procedures, and treatments delivered to neurovascular patients within the NVNs. The analysis included case numbers for stroke, thrombolytic therapies, thrombectomies, intracerebral hemorrhages, subarachnoid hemorrhages, and carotid interventions at both coordinating centers and network hospitals. Process times—door-to-needle and door-to-groin puncture times—were systematically recorded. Data were derived from the 15 NVNs certified at that time, with parameters dating back mainly to the years 2017 to 2019.

The present article updates these findings, analyzing audit reports from 19 NVNs evaluated and certified between 2021 and 2024 (reflecting data from 2020 to 2023). Recent developments and advances in neurovascular patient care within NVNs are addressed, as well as ongoing challenges. Importantly, treatment and procedural parameters between coordinating centers and partner hospitals are analyzed and compared for the 2020–2023 period.

## Material and methods

Data were extracted from audit reports of 19 neurovascular networks (NVNs) certified between 2021 and 2024. The parameters reported during the audit reflect the year preceding the audit, i.e., correspond to the period from 2020 to 2023. Audit reports were self-reported by the respective hospitals, and reviewed by independent auditors as part of the certification process. The certification of NVNs was developed by the German societies of Stroke, Neurosurgery, and Neuroradiology. It is based on detailed quality criteria (structural, processual) for both the coordinating centers and partner hospitals, which are regularly evaluated through on-site audits conducted on a three-year cycle (see https://www.dsg-info.de/stroke-units-neurovaskulaere-netzwerke/neurovaskulaere-netzwerke/).

Following Röther et al. [12], mean values and ranges were calculated for the following parameters:

*Coordinating centers:* Number of stroke patients treated within the NVNs and at the coordinating centers; number of stroke unit (SU) beds per coordinating center; existence of an independent neuro-intensive care unit; number of i.v. thrombolyses administered per coordinating center; median door-to-needle time; number of thrombectomies performed at the coordinating centers; median door-to-groin puncture time (minutes); number of intracerebral hemorrhages (ICH) per coordinating center; number of surgically treated ICHs per coordinating center; number of aneurysmal subarachnoid hemorrhages (SAH) per coordinating center; number of carotid stenting procedures per coordinating center; number of carotid endarterectomies per coordinating center. For NVNs with more than one coordinating center (one NVN with two, one with three), data were aggregated, as these parameters were not always reported separately in the audit documentation.

*Partner / network hospitals:* Total number of partner hospitals; number of partner hospitals per NVN; distance to the coordinating center; number of stroke patients treated in the partner hospitals; proportion of regional stroke units among partner hospitals; proportion of supra-regional stroke units among partner hospitals; proportion of non-certified hospitals among partner hospitals; number of patients transferred to the coordinating center; number of patients transferred per NVN; number of patients transferred for thrombectomy; number of patients transferred with intracerebral hemorrhage (ICH); number of patients transferred with subarachnoid hemorrhage (SAH); number of patients transferred for carotid stenting or surgery; number of patients transferred to the coordinating center for other reasons (e.g., decompressive hemicraniectomy, treatment of vascular malformations).

The results obtained from the certification process of 19 NVNs in 2021–2024 were compared with those reported by Röther et al. [12], which were based on data of 15 NVNs from 2017–2019, to assess recent developments in neurovascular care within the NVNs.

## Results

Of the 19 NVNs, 14 have a university hospital and 5 a non-university comprehensive care hospital serving as the coordinating center. One NVN has two coordinating centers, and another has three; for these, data from coordinating centers were summed. In total, the NVNs included 148 partner hospitals (compared to 107 in 2017–2019), with the number of partner hospitals per NVN ranging from 3 to 16. Collectively, the NVNs treated 119,907 stroke patients per year, reflecting an increase of approximately 33,400 cases (+ 39%) as compared to the 2017–2019 period, coinciding with the expansion from 15 to 19 NVNs. The average distance between partner hospitals and their coordinating center was 40 km (range: 2–143 km), and a total of 3,626 patients were transferred between partner hospitals and coordinating centers. Table 1 shows how parameters reflecting the treatment of neurovascular patients within the NVNs developed from the 2017–2019 to the 2020–2023 period. Table 2 shows procedures performed in coordinating centers and partner hospitals during the 2020–2023 period.

**Table 1:** Neurovascular networks in Germany: Comparison of data from 15 certified neurovascular networks, primarily from 2017 to 2019 [12], with data from 19 certified neurovascular networks, from 2020–2023

	2017–2019	2020–2023
*NVN coordinating centers*		
Total strokes treated in NVNs	86,510	119.907
Strokes treated in coordinating centers	18.206	26.235
Strokes per coordinating center	1,214 (895–1,635)	1,381 (810–3,490)
Stroke unit (SU) beds per coordinating center	13 (8–18)	14 (8–36)
Independent neuro-ICU	08/15	10/19
I.v. Thrombolyses administered per coordinating center	170 (40–384)	172 (83–370)
Door-to-needle time (minutes)	32 (23–42)	34 (26–41)
Total thrombectomies	2.463	4.488
Thrombectomies per coordinating center	164 (79–387)	236 (103–537)
Door-to-groin time (minutes)	65 (47–85)	73 (46–87)
ICH cases per coordinating center	159 (36–384)	204 (22–487)
ICH cases undergoing surgery per coordinating center	52 (12–123)	56 (0–143)
Carotid stenting per coordinating center	75 (9–456)	79 (24–190)
Carotid endarterectomy per coordinating center	83 (34–184)	76 (21–209)
*NVN partner hospitals*		
Total number of partner hospitals	107	148
Partner hospitals per NVN	7 (3–16)	8 (3–16)
Distance to coordinating center (km)	25 (4–147)	40 (2–143)
Strokes treated in partner hospitals	64.158	93.672
Strokes per partner hospital	625 (28–1,737)	642 (15–1,832)
Regional stroke units/total partner hospitals	69/107	80/148
Supraregional stroke units/total partner hospitals	6/107	26/148
Non-certified hospitals/total partner hospitals	32/107	42/148
Total patient transfers to coordinating center	2.726	3.626
Transferred patients per NVN	182 (64–541)	191 (46–361)
Transferred for thrombectomy	1.015	1.971
Transferred for ICH	579	571
Transferred for aneurysmal SAH	373	439
Transferred for carotid stenting or endarterectomy	268	439
Other transfers	420	206

ICH intracerebral hemorrhage, NVN neurovascular network, SAH subarachnoid hemorrhage, SU stroke unit, ICU intensive care unit

Presented are mean values per year; numbers in parentheses represent range

**Table 2:** Procedures performed in NVN coordinating centers and NVN partner hospitals

	coordinating centers	partner hospitals
I.v. thrombolysis, per center/hospital*	172 (83–370)	89 (3–331)
I.v. thrombolysis to stroke ratio (ischemic + hemorrhagic)**	12.6%	12.4%
I.v. thrombolysis to ischemic stroke ratio	16.1%	
Thrombectomy, per center/hospital*	236 (103–537)	59 (1–211)
Thrombectomy to stroke ratio (ischemic + hemorrhagic)**	17.3%	4.1%
Thrombectomy to ischemic stroke ratio	22.1%	
Carotid stenting, per center/hospital*	79 (24–190)	19 (1–98)
Carotid endarterectomy, per center/hospital*	76 (21–209)	54 (1–193)
Aneurysm coiling (acute), per center/hospital*	40 (21–133)	14 (2–27)
Aneurysm coiling (elective), per center/hospital*	63 (18–179)	19 (1–61)
Aneurysm clipping (acute), per center/hospital*	25 (6–89)	12 (1–43)
Aneurysm clipping (elective), per center/hospital*	26 (4–82)	6 (1–24)

^*^data for partner hospitals performing ≧1 of the respective procedure(s) per year

^**^data for all partner hospitals

Presented are mean values per year; numbers in parentheses represent range

### NVN coordinating centers

The coordinating centers collectively treated 26,235 stroke patients annually, with an average of 14 stroke unit beds per center. This represents an increase of approximately 8,000 stroke cases (+ 44%) compared to the previous period, as the number of NVNs increased from 15 to 19. The average number of strokes treated per coordinating center increased from 1,214 to 1,381 per year. 10 of the 19 centers operated an independent neurological and/or neurosurgical intensive care unit. The coordinating centers performed an average of 172 (range: 83–370) systemic thrombolysis procedures per year, as compared to 170 per year in the previous period. The i.v. thrombolysis to ischemic stroke ratio was 16.1% (range: 8.0% to 32.4%), and the i.v. thrombolysis to all stroke ratio (ischemic and hemorrhagic) was 12.6% (range: 7.1% to 23.9%). A total of 4,488 mechanical thrombectomies were performed (236 per coordinating center, range: 103–537), representing a substantial increase as compared to the 2017–2019 period (total increase: + 2,025 thrombectomies [+ 82%]; per center: + 72 thrombectomies [+ 44%]). The thrombectomy to ischemic stroke ratio was 22.1% (range: 10.5%-33.7%); the thrombectomy to all stroke ratio (ischemic and hemorrhagic) was 17.3% (range: 8.4%-29.4%). On average, 5 neurointerventionalists per center performed thrombectomy and endovascular aneurysm treatment procedures (modules E and F; range: 2–18).

The median door-to-needle time was 34 min (range: 26–41), and the median door-to-groin time was 73 min (range: 46–87), both showing a slight increase compared to 2017–2019. However, only 14 of 19 centers reported median door-to-needle times, and only 12 of 19 provided median door-to-groin times; for the remaining centers, only percentage values (> 30%) were provided, and were not included in the analysis. A total of 3,876 patients with non-traumatic intracerebral hemorrhage (ICH) were treated, of whom 1,072 underwent surgical intervention (mean: 204 ICH cases per coordinating center, range: 22–487; 56 surgically treated cases, range: 0–143).

A total of 2,946 patients underwent aneurysm treatment, including acute clipping (mean: 25 per coordinating center, range: 6–89), elective clipping (mean: 26 per coordinating center, range: 4–82), acute coiling (mean: 40 per coordinating center, range: 21–133), and elective coiling (mean: 63 per coordinating center, range: 18–179). The mean clipping-to-coiling ratio was 0.7 (range: 0.13–2.95). Carotid artery interventions included: 1,412 carotid stenting procedures (mean: 79 per coordinating center, range: 24–190) and 1,360 carotid endarterectomies (mean: 76 per coordinating center, range: 21–209).

### NVN partner hospitals

The 148 NVN partner hospitals treated a total of 93,672 stroke patients per year (mean: 642 per hospital, range: 15–1,832), representing an increase of approximately 29,000 cases (+ 46%) compared to the previous period. Of the partner hospitals, 80 had a regional stroke unit, 26 had a supra-regional stroke unit, and 42 either had a non-certified stroke unit, an internal medicine department providing stroke care, or did not specify their status. On average, 191 patients (range: 46–361) per NVN were transferred annually between partner hospitals and the coordinating center. Transfers included 1,971 patients for mechanical thrombectomy, 571 for treatment of intracerebral hemorrhage, 439 for treatment of aneurysmal subarachnoid hemorrhage, 439 for carotid stenting or carotid endarterectomy, and 206 for other reasons, such as decompressive hemicraniectomy or treatment of vascular malformations. The number of patients transferred for mechanical thrombectomy nearly doubled compared to the 2017–2019 period, increasing from 1,015 to 1,971 cases.

Of the 148 partner hospitals, 111 performed intravenous thrombolysis (2 did not; 34 provided no information, many of which likely do not perform thrombolysis). Among those performing intravenous thrombolysis, the mean was 89 procedures annually per hospital (range: 3–331). The i.v. thrombolysis to stroke ratio (ischemic and hemorrhagic) was 12.4% (range: 0.0% to 27.6%); calculation for ischemic strokes alone was not feasible due to insufficient reporting of stroke subtype proportions for partner hospitals. 57 partner hospitals performed mechanical thrombectomy (36 did not; 55 provided no information). Among those performing the procedure, the mean was 59 thrombectomies annually per hospital (range: 1–211). The thrombectomy to stroke ratio (ischemic and hemorrhagic) was 4.1% (range: 0.0%-24.1%).

20 partner hospitals performed acute aneurysm clipping (52 did not; 76 provided no information). Among those performing the procedure, the mean was 12 acute clippings annually per hospital (range: 1–43). 16 partner hospitals performed elective aneurysm clipping (53 did not; 79 provided no information). Among those performing the procedure, the mean was 6 elective clippings annually per hospital (range: 1–24). 15 partner hospitals performed acute aneurysm coiling (43 hospitals did not; 63 provided no information). Among those performing the procedure, the mean was 14 acute coilings annually per hospital (range: 2–27). 19 partner hospitals performed elective aneurysm coiling (40 hospitals did not; 62 provided no information). Among those performing the procedure, the mean was 19 elective coilings annually per hospital (range: 1–61). For partner hospitals performing both procedures, the mean clipping-to-coiling ratio was 1.3 (range: 0.05–6.9).

52 partner hospitals performed carotid stenting (32 did not; 64 provided no information). Among those performing the procedure, the mean was 19 carotid stentings annually per hospital (range: 1–98). 79 partner hospitals performed carotid endarterectomy (15 did not; 54 provided no information). Among those performing the procedure, the mean was 54 carotid endarterectomies annually per hospital (range: 1–193).

**Fig. 2:**
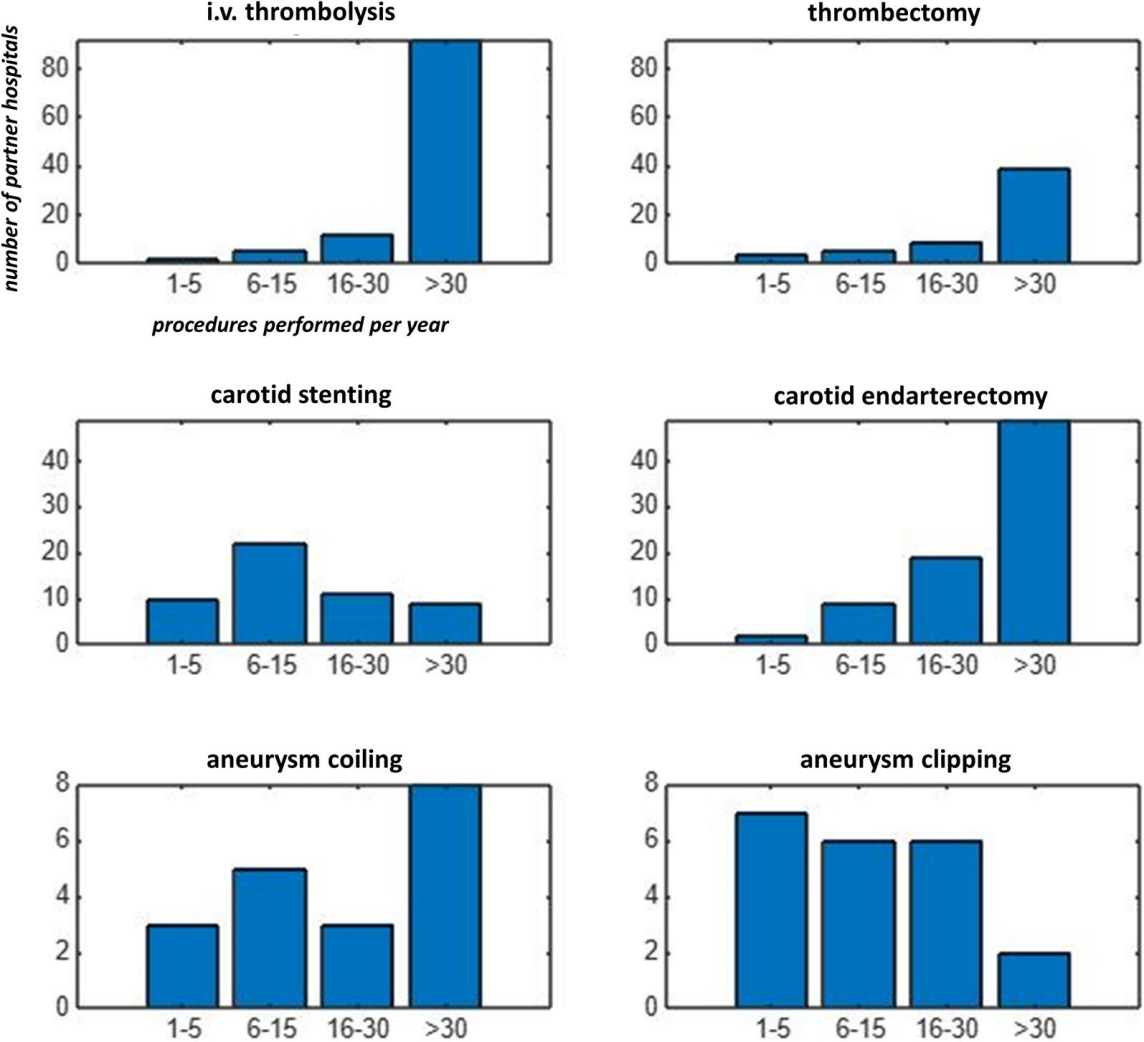
Bar graphs showing the numbers of procedures performed by partner hospitals per year, for i.v. thrombolysis, thrombectomy, carotid stenting, carotid endarterectomy, aneurysm coiling, and aneurysm clipping

Further analysis of procedure volumes among partner hospitals is shown in Fig. 2: Intravenous thrombolysis: 2 hospitals performed 1–5; 5 performed 6–15; 12 performed 16–30; and 92 performed more than 30 procedures per year. Mechanical thrombectomy: 4 hospitals performed 1–5; 5 performed 6–15; 9 performed 16–30; and 39 performed more than 30 procedures per year. Carotid stenting: 10 hospitals performed 1–5; 22 performed 6–15; 11 performed 16–30; and 9 performed more than 30 procedures per year. Carotid endarterectomy: 2 hospitals performed 1–5; 9 performed 6–15; 19 performed 16–30; and 49 performed more than 30 procedures per year. Aneurysm coiling: 3 hospitals performed 1–5; 5 performed 6–15; 3 performed 16–30; and 8 performed more than 30 procedures per year. Aneurysm clipping: 7 hospitals performed 1–5; 6 performed 6–15; 6 performed 16–30; and 2 performed more than 30 procedures per year.

## Discussion

The present data provide an updated overview of neurovascular care delivered in 22 coordinating centers and 148 partner hospitals within 19 certified neurovascular networks (NVNs) in Germany between 2020 and 2023. Annually, approximately 120,000 stroke patients were treated within these NVNs. Given an estimated total of 262,000 strokes per year in Germany [6], nearly one in two stroke patients received care in a certified NVN, underscoring the central role of these networks in acute stroke management. The results demonstrate the continued development of NVN structures in recent years. In particular, the expansion from 15 to 19 NVNs, and from 107 to 148 partner hospitals, has led to an increase in annual stroke cases managed in coordinating centers (from 18,203 to 26,235), total strokes treated within NVNs (from 86,510 to 119,907), total mechanical thrombectomies (from 2,463 to 4,488), thrombectomies per coordinating center (from 164 to 236), and transfers for thrombectomy from partner hospitals to coordinating centers (from 1,015 to 1,971). These findings highlight the rising significance and prevalence of mechanical recanalization therapies for ischemic stroke within the NVN structures, reflecting national trends toward increased access to endovascular treatment.

In contrast, the number of systemic thrombolyses per coordinating center remained largely unchanged as compared to the previous period (170 to 172 annually), reflecting that intravenous thrombolysis is a well-established therapy for acute ischemic stroke [13]. Still, opposing effects may influence these figures: while intravenous thrombolysis may be administered more frequently when indicated, other factors—such as increased prehospital prescription of anticoagulation, e.g. in atrial fibrillation, leading to more frequent contraindications—could counterbalance this trend.

Process times showed a slight increase, with median door-to-needle times rising from 32 to 34 min and door-to-groin puncture times from 65 to 73 min. However, the interpretation of door-to-groin times is limited. It can be lowered if a coordinating center is receiving more patients from a partner hospital, since imaging is performed in the primary hospital. Importantly, only 14 out of 19 coordinating centers reported a median door-to-needle time, and only 12 reported a median door-to-groin time (for other centers, only ranges and percentages, e.g., XX% < 30min and YY% < 60min were reported). Consequently, only these data could be included in the analysis. More generally, differences in NVN composition over time (15 to 19 NVNs) must also be considered when interpreting the results. Also, the Covid-19 pandemic and the associated infection control measures have affected stroke management in Germany [2], and may have potentially prolonged process times during the 2020–2023 time period. Finally, imaging protocols have been advanced in this period that allowed for a quantification of tissue at risk (DAWN, [9],DEFUSE-3, [1]). This enabled physicians to treat more patients than previously, possibly contributing to the overall increase in intraarterial therapies. At the same time, the additional imaging could have increased door to groin times due to the additional time for acquisition, processing, analysis, and interpretation.

In sum, NVNs are well established and play a crucial role in providing high-quality care to neurovascular patients. However, as their distinct benefit remains empirically to be defined, future studies should focus on the additional value of NVNs as compared to stand-alone structures with respect to the quality of interdisciplinary stroke care.

Comparative analysis of coordinating centers and partner hospitals for the 2020–2023 period revealed considerable heterogeneity among partner hospitals. Among them are large hospitals operating supra-regional stroke units, having independent neuroradiology and neurosurgery departments, and treating more than 1,500 stroke patients per year. But partner hospitals also include internal medicine departments managing a small number of stroke cases annually. Notably, missing data were frequent among partner hospitals (up to 50% for some procedures); but this likely reflects the absence of such services at those sites rather than incomplete reporting.

When comparing thrombolysis and thrombectomy procedures in coordinating centers and partner hospitals, data indicate that i.v. thrombolysis is performed equally in coordinating centers and partner hospitals, with a thrombolysis to stroke ratio (ischemic and hemorrhagic) at around 12% (16% for ischemic strokes in coordinating centers), whereas patients eligible for thrombectomy concentrate in the coordinating centers, with a thrombectomy to stroke ratio of 17% vs. 4% (22% for ischemic strokes in coordinating centers). These parameters meet the targets for the development of stroke care for 2030, as formulated by the Action Plan for Stroke in Europe (i.e., i.v. thrombolysis rates above 15% and thrombectomy rates above 5%; [10]).

Next to thrombectomy, the centralization of high-expertise procedures for the treatment of hemorrhagic stroke—i.e., surgical management of ICH, and clipping or coiling for aneurysmal SAH—within coordinating centers is an explicit goal of the NVN structure (as significant procedural volume-outcome effects, e.g., in ruptured intracranial aneurysm treatment, have been described; [5]). This intended concentration of care, however, yet requires empirical validation in longitudinal studies. Importantly, absolute numbers of advanced procedures, including thrombectomy, carotid stenting, carotid endarterectomy, and aneurysm coiling or clipping, were considerably lower in partner hospitals, with some institutions performing only a few of these complex interventions annually. This raises questions regarding procedural standards and quality of care for these procedures, and strict limitations from regulatory sites are mandatory.

## Conclusion

Certified neurovascular networks (NVNs) have become established within the German healthcare system in recent years, and play a pivotal role in providing high-quality care to patients with neurovascular diseases. Ongoing advances in infrastructure and process quality, as well as the increasing and widespread implementation of mechanical thrombectomy, underscore the effectiveness of the NVN structures. The consolidation of specialized expertise and the increased visibility of NVNs in Germany have further strengthened the overall neurovascular field.

## Data Availability

The raw data supporting the conclusions of this article will be made available by the authors, without undue reservation.
